# Impact of adverse events, treatment modifications, and dose intensity on survival among patients with advanced renal cell carcinoma treated with first-line sunitinib: a medical chart review across ten centers in five European countries

**DOI:** 10.1002/cam4.302

**Published:** 2014-07-18

**Authors:** Camillo Porta, Antonin Levy, Robert Hawkins, Daniel Castellano, Joaquim Bellmunt, Paul Nathan, Ray McDermott, John Wagstaff, Paul Donnellan, John McCaffrey, Francis Vekeman, Maureen P Neary, Jose Diaz, Faisal Mehmud, Mei Sheng Duh

**Affiliations:** 1 IRCCS San Matteo University Hospital FoundationPavia, Italy; 2 Institut Gustave RoussyParis, France; 3 School of Cancer and Imaging Sciences, University of ManchesterManchester, United Kingdom; 4 Hospital 12 de OctubreMadrid, Spain; 5 Hospital del Mar-IMIMBarcelona, Spain; 6 Mount Vernon Cancer CentreNorthwood, Middlesex, United Kingdom; 7 Adelaide & Meath HospitalTallaght, Dublin, Ireland; 8 South West Wales Cancer Institute, Singleton HospitalSwansea, United Kingdom; 9 University College Hospital GalwayGalway, Ireland; 10 Mater Misericordiae HospitalDublin, Ireland; 11 Groupe d'analyse, LtéeMontréal, Québec, Canada; 12 GlaxoSmithKlineCollegeville, Pennsylvania; 13 Analysis Group, Inc.Boston, Massachusetts

**Keywords:** Angiogenesis, clinical observations, statistical methods, urological oncology

## Abstract

Angiogenesis inhibitors have become standard of care for advanced and/or metastatic renal cell carcinoma (RCC), but data on the impact of adverse events (AEs) and treatment modifications associated with these agents are limited. Medical records were abstracted at 10 tertiary oncology centers in Europe for 291 patients ≥18 years old treated with sunitinib as first-line treatment for advanced RCC (no prior systemic treatment for advanced disease). Logistic regression models were estimated to compare dose intensity among patients who did and did not experience AEs during the landmark periods (18, 24, and 30 weeks). Cox proportional hazard models were used to explore the possible relationship of low-dose intensity (defined using thresholds of 0.7, 0.8, and 0.9) and treatment modifications during the landmark periods to survival. 64.4% to 67.9% of patients treated with sunitinib reported at least one AE of any grade, and approximately 10% of patients experienced at least one severe (grade 3 or 4) AE. Patients reporting severe AEs were statistically significantly more likely to have dose intensities below either 0.8 or 0.9. Dose intensity below 0.7 and dose discontinuation during all landmark periods were statistically significantly associated with shorter survival time. This study of advanced RCC patients treated with sunitinib in Europe found a significant relationship between AEs and dose intensity. It also found correlations between dose intensity and shorter survival, and between dose discontinuation and shorter survival. These results confirm the importance of tolerable treatment and maintaining dose intensity.

## Background

Renal cell carcinoma (RCC) is the most common cancer of the kidney, with 30% of patients presenting with metastatic disease. Because RCC is highly resistant to chemotherapy, treatment options have been limited. Cytokines (e.g., interleukin-2 or interferon alpha) have been widely used as first-line treatment for advanced or metastatic RCC, but have low-response rate, coupled with relevant toxicity; furthermore, they do not have a survival benefit for patients with disease of intermediate prognosis [1]. More recently, antiangiogenesis agents have been used as alternative treatment. One such agent, Sutent® (sunitinib malate), received accelerated approval from the Food and Drug Administration (FDA) for treatment of advanced RCC in January 2006 [2].

However, safety data from both clinical trials and the expanded access program (EAP) for sunitinib demonstrated that adverse events (AEs) are common among patients undergoing this treatment [3–5]. More recent data suggest an emerging role for immunotherapy using the new approach targeting the immune check point in RCC [6].

The objective of this study was to assess the relationships between AEs and treatment patterns as well as between treatment patterns and survival among patients with advanced RCC receiving first-line sunitinib in real-world clinical practice. First, we tested whether there is an association between AEs and dose intensity among patients receiving sunitinib as first-line treatment. Second, we investigated whether an association exists between dose intensity and overall survival (OS). Third, we measured the association between treatment modifications due to AEs and OS.

## Methods

### Study design

A retrospective, open-cohort study was conducted using data from medical records for a total of 291 eligible patients with advanced RCC who received sunitinib in major institutions with expertise in treatment of advanced RCC across five European countries. Patients had no prior systemic treatment for advanced disease. The observation period for each patient started from the date of first sunitinib prescription to the earliest of date of death, last follow-up date at the clinic, or date of medical record abstraction. Patients continued to be followed if they switched to any second-line or third-line anti-angiogenesis agents.

### Study population

To become eligible for the study patients were required to meet the following inclusion criteria: (1) have had a confirmed histological and/or cytological diagnosis of locally advanced or metastatic RCC; (2) 18 years old or older at the time of confirmed diagnosis of advanced RCC; (3) received at least 1 dose of oral sunitinib after 1 January 2005; (4) were treatment naïve for advanced disease (including angiogenesis inhibitor-naïve) prior to receiving the first prescription for sunitinib, and (5) were actively treated at the clinic to ensure complete longitudinal data. Patients were excluded if their first angiogenesis inhibitor treatment was initiated less than 3 months prior to the start date of medical record data abstraction, which varied across sites, to ensure adequate follow-up time.

### Data source

Local institutional review board/ethics committee approval was sought and obtained for the collection of data from medical charts from 10 major treatment centers for advanced RCC in five European countries. Data for this study were derived from medical charts reviewed for treatment provided between July 2008 and December 2010 from oncology treatment centers in France (Institut Gustave Roussy), Ireland (Adelaide and Meath Hospital Tallaght, Mater Misericordiae University Hospital, and University College Hospital Galway), Italy (IRCCS Policlinico San Matteo), Spain (Hospital Universitario 12 de Octubre, and Hospital del Mar), and the United Kingdom (Mount Vernon Cancer Centre, South West Wales Cancer Institute, and Christie Hospital NHS Trust). Medical records from all patients who met the study criteria and who had archived and accessible records were included in the study. Medical records were retrospectively reviewed and extracted by clinical personnel.

Data extracted from the medical records included but were not limited to: date of initial advanced RCC diagnosis, demographic variables, comorbidities, prior pharmacological or radiological treatments, metastatic site(s), drug-related adverse event data, laboratory data, and radiologic test results. The dates of treatment initiation and discontinuation, initial dosing, dates and reasons for treatment interruptions and treatment changes, dosing modifications, and follow-up tumor assessments were also recorded.

### Landmark periods

This study uses landmark analysis to assess the relationship between treatment patterns and overall survival. A landmark period is defined as a period of time following treatment initiation during which an exposure of interest is observed (e.g., AEs). Patients are then divided between those with and without the exposure during the landmark period and outcomes are compared from the end of the landmark period until the end of observation. In the current study, a landmark period of 24 weeks following sunitinib treatment initiation was used and sensitivity analyses using 18 and 30 weeks were also considered.

### Event definitions

#### Adverse events

Study investigators at the clinics retrospectively assessed toxicity experienced by patients while taking sunitinib and graded the AEs using the National Cancer Institute Common Terminology Criteria for AEs (CTCAE) version 3.0 [7]. If the severity of the AE was unknown then grade 1 was assigned. Only AEs experienced by patients during their first-line sunitinib treatment were considered for the assessment of safety.

#### Treatment modifications due to AEs

Treatment modifications included dose reductions, treatment interruptions (temporary stoppage of treatment with intent to resume treatment), and treatment discontinuations. Reasons for treatment modifications were also abstracted from patients' medical records, and only modifications that were responses to AEs were considered in this analysis. Survival as measured from the end of the landmark period was then compared between patients with and without treatment modifications during the landmark period.

#### Dose intensity

Dose intensity was calculated as the actual daily dose of sunitinib received by patients divided by the optimal or recommended daily dose. For example, suppose a patient received a total of four cycles of 4 weeks treatment followed by 2 weeks off (4/2), but after two cycles at 50 mg/day, the dose was reduced to 37.5 mg/day. This patient's actual daily dose was 29.7 mg instead of the 33.3 mg recommended daily dose. The relative dose intensity of the patient over these four cycles was therefore 89.1% [(29.7/33.3) × 100]. In addition to dose reductions and dose interruptions, changes in treatment schedules, such as moving from a continuous or 5/1 schedule to a 4/2 schedule would influence dose intensity.

The impact of three thresholds of low-dose intensity was explored: <70%, <80%, and <90%; these cutoffs were prespecified as part of the study protocol. For the study of the association between AEs and dose intensity, dose intensity was measured from the end of the AE observation period (equivalent to the landmark period) until the end of observation. For the study of the association between dose intensity and OS, dose intensity was measured during the landmark period. Dose intensity for the entire observation period was not considered in either analysis given the study design and the need to assess dose intensity as the outcome from the end of the AE observation period for the analysis of the association between AEs and dose intensity or as the exposure during the landmark period for the analysis of the association between dose intensity and OS.

#### Overall survival

Time to death was defined as the time from the end of the landmark period to the date of death. Patients who did not die by the study end date were censored at the date of last follow-up. For the study of the association between AEs and dose intensity, patients who died or had their last follow-up date during the landmark period were dropped from the sample analyzed. Similarly, for the OS analyses, patients who died or had their last follow-up date during the landmark period were dropped from the sample analyzed.

### Statistical analysis

Descriptive statistics were used to describe patient baseline characteristics, AEs, dose intensity, and treatment modifications. Means, median, and ranges were used to describe continuous variables; frequencies and percentages were reported for categorical variables. The association between AEs and different dose intensity thresholds was assessed using a multivariate logistic regression model. In addition to the indicator for AEs during the AE observation period, other covariates considered in the model included age at treatment initiation, number of metastatic sites, gender, time from initial RCC diagnosis to initiation of first-line sunitinib treatment, and country. The strength of association between dose intensity or treatment modifications and time to death was explored using multivariate Cox proportional hazards regression models. The Cox regressions included the same controls as the logistic regression model. All analyses were performed using SAS software version 9.2 (SAS Institute, Inc., Cary, NC).

## Results

### Patient characteristics

A total of 291 patients in France (*n* = 65), Ireland (*n* = 53), Italy (*n* = 15), Spain (*n* = 39), and the UK (*N* = 119) received first-line sunitinib and met the eligibility criteria for this study. Table 1 presents baseline characteristics of the study population.

**Table 1:** Baseline characteristics among patients with advanced RCC treated with sunitinib as first-line angiogenesis inhibitor treatment.

	Patients receiving sunitinib
	*N* = 291
Age at treatment initiation, years	
Median (range)	62.2 (25.9–88.6)
Mean (SD)	60.9 (12.0)
Male, *n* (%)	196 (67.4)
Number of metastatic sites, *n* (%)	
0	16 (5.5)
1	124 (42.6)
2	89 (30.6)
≥3	61 (21.0)
Unknown	1 (0.3)
Country of treatment, *n* (%)	
France	65 (22.3)
Italy	15 (5.2)
Ireland	53 (18.2)
UK	119 (40.9)
Spain	39 (13.4)
Time from initial RCC diagnosis to treatment, months	
Mean (SD)	26.0 (39.7)
<1 year, *n* (%)	162 (55.7)

RCC, renal cell carcinoma; SD, standard deviation.

### Adverse events and dose intensities

Table 2 reports summary statistics on AEs and dose intensities. Of the 184 patients with a sunitinib treatment duration of at least 24 weeks, 125 (67.9%) reported at least one AE of any grade, and 19 (10.3%) had at least one grade 3 or 4 AE.

**Table 2:** Summary of adverse events and dose intensity among patients with advanced RCC treated with sunitinib as first-line angiogenesis inhibitor treatment.

	AE observation period (landmark period)		
	
	18 weeks	24 weeks	30 weeks
			
Patients with treatment duration exceeding the AE observation period	*N* = 205	*N* = 184	*N* = 156
All grades AEs			
≥1 AE, *n* (%)^1^	132 (64.4)	125 (67.9)	109 (69.9)
Most frequent AEs, *n* (%)			
Fatigue/Asthenia	58 (28.3)	58 (31.5)	54 (34.6)
Mucositis/Stomatitis	51 (24.9)	51 (27.7)	51 (32.7)
Diarrhea	33 (16.1)	33 (17.9)	33 (21.2)
Nausea	27 (13.2)	26 (14.1)	26 (16.7)
Hand–foot syndrome	18 (8.8)	20 (10.9)	20 (12.8)
Skin rash	12 (5.9)	15 (8.2)	15 (9.6)
Grades 3 and 4 AEs			
≥1 AE, *n* (%)^2^	19 (9.3)	19 (10.3)	14 (9.0)
Most frequent AEs, *n* (%)			
Fatigue/Asthenia	5 (2.4)	5 (2.7)	5 (3.2)
Mucositis/Stomatitis	5 (2.4)	4 (2.2)	2 (1.3)
Diarrhea	2 (1.0)	2 (1.1)	2 (1.3)
Hand–foot syndrome	1 (0.5)	2 (1.1)	2 (1.3)
Pain	1 (0.5)	1 (0.5)	1 (0.6)
Thrombotic events	1 (0.5)	1 (0.5)	1 (0.6)
Duration of treatment beyond the AE observation period, weeks, mean (SD)	44.9 (58.5)	43.8 (59.9)	44.9 (62.2)
Dose intensity^3^^,^^4^, mean (SD)			
All grades AEs			
No AE	0.887 (0.232)	0.891 (0.221)	0.878 (0.204)
≥1 AE	0.843 (0.253)	0.837 (0.250)	0.844 (0.243)
Grades 3 and 4 AEs			
No AE	0.869 (0.248)	0.869 (0.239)	0.872 (0.224)
≥1 AE	0.771* (0.205)	0.748* (0.230)	0.689 (0.214)
Patients with low-dose intensity, *n* (%)^3^			
<0.9	73 (35.6)	69 (37.5)	60 (38.5)
<0.8	64 (31.2)	60 (32.6)	50 (32.1)
<0.7	38 (18.5)	35 (19.0)	28 (17.9)

AE, adverse event; RCC, renal cell carcinoma; SD, standard deviation.

1 Includes all patients with at least 1 AE during the respective AE observation period.

2 Includes all patients with at least one grade 3/4 AEs during the respective AE observation period.

3 Dose intensity calculated over the duration of treatment following the respective AE observation period.

4 The “*” indicates that the mean dose intensity is statistically significantly different between the no AE and ≥1 AE groups at an *α* level of 0.05.

The average duration of sunitinib treatment beyond the 24-week observation period was 43.8 weeks. Mean dose intensities were significantly different among patients who did and did not report ≥1 grade 3 or 4 AE (≥1 grade 3 or 4 AE: 19; 0 grade 3 or 4 AE: 165, mean dose intensity: 0.748 vs. 0.869, 95% confidence interval [CI] of difference: [0.006, 0.237]), but were not significantly different among patients who did and did not report ≥1 all grade AE (≥1 AE: 125; 0 AE: 59, mean dose intensity: 0.837 vs. 0.891, 95% CI of difference: [−0.013, 0.121]). Of the 184 patients observed during the 24-week AE observation period, 69 (37.5%), 60 (32.6%), and 35 (19.0%) had dose intensities below 0.9, 0.8, and 0.7, respectively, for the duration of treatment following the 24-week period.

Table 3 presents adjusted odds ratios quantifying the strength of association between AEs and sunitinib low-dose intensity. There was no statistically significant association between the development of an AE of any grade within 24 weeks of treatment initiation and low-dose intensity treatment following this period for all three thresholds used to define low-dose intensity. Patients with ≥1 grade 3 or 4 AE during the first 24 weeks of treatment were 5.12 (95% CI: [1.27, 20.68]) times more likely to have a dose intensity below 0.8 and 6.79 (95% CI: [1.39, 33.26]) times more likely to have a dose intensity below 90% following the AE observation period. Adverse event observation periods of 18 and 30 weeks produced similar results for both any grade and grade 3 or 4.

**Table 3:** Association between adverse events and low-dose intensity in patients with advanced RCC treated with sunitinib as first-line angiogenesis inhibitor treatment[Table-fn tf3-2].

	Odds ratio (95% CI)^2^		
	
	Low-dose intensity <0.7	Low-dose intensity <0.8	Low-dose intensity <0.9
All Grade AEs (reference: no AE)			
AE within 18 weeks of treatment initiation	1.60 (0.65, 3.98)	1.56 (0.75, 3.24)	1.60 (0.80, 3.21)
AE within 24 weeks of treatment initiation	2.40 (0.80, 7.19)	1.71 (0.76, 3.83)	1.63 (0.76, 3.50)
AE within 30 weeks of treatment initiation	2.14 (0.63, 7.24)	1.83 (0.76, 4.40)	2.10 (0.90, 4.88)
Grades 3 and 4 AEs (reference: no grade 3/4 AE)			
AE within 18 weeks of treatment initiation	5.12 (1.33, 19.71)	7.19 (1.44, 35.86)	5.61 (1.12, 28.09)
AE within 24 weeks of treatment initiation	3.18 (0.89, 11.36)	5.12 (1.27, 20.68)	6.79 (1.39, 33.26)
AE within 30 weeks of treatment initiation	2.09 (0.59, 7.39)	4.97 (1.39, 17.72)	8.75 (1.81, 42.21)

AE, adverse event; CI, confidence interval; RCC, renal cell carcinoma.

1 Each model was adjusted for age at treatment initiation, number of metastatic sites, gender, time from initial RCC diagnosis to treatment, and country.

2 Confidence intervals are computed with robust standard errors clustered at the country level.

### Dose intensity and overall survival

Table 4 summarizes results from multivariate Cox proportional hazard models assessing the association between sunitinib dose intensity and survival. Of the 291 patients included in this study, 217 patients were taking sunitinib and had not died at the end of the 24-week landmark period. Sixty four (29.5%) of these patients experienced at least one treatment modification (dose reduction or treatment interruption) that led to a dose intensity below 0.9 during the landmark period. Dose intensities below 0.8 and 0.7 during the 24-week landmark period were observed in 34 (15.7%) and 15 (6.9%) patients, respectively. The predominant initial dosing schedule for sunitinib was 50 mg QD 4/2; overall, 81% of patients initiated treatment with this schedule and the proportion that initiated at this dose and schedule varied from 66% to 100% in the five countries (data not shown).

**Table 4:** Association between low-dose intensity and time to death in patients with advanced RCC treated with sunitinib as first-line angiogenesis inhibitor treatment^1^.

Dose intensity during landmark period	Dose intensity observation period (landmark period): 0–18 weeks		Dose intensity observation period (landmark period): 0–24 weeks		Dose intensity observation period (landmark period): 0–30 weeks	
		
	*N* = 236		*N* = 217		*N* = 195	
Low-dose Intensity <0.90	Yes: 64	No: 172	Yes: 64	No: 153	Yes: 59	No: 136
HR (95% CI) (reference: dose intensity ≥0.90)	0.94 (0.56, 1.58)		0.96 (0.56, 1.65)		1.30 (0.74, 2.29)	
Low-dose Intensity <0.80	Yes: 33	No: 203	Yes: 34	No: 183	Yes: 37	No: 158
HR (95% CI) (reference: dose intensity ≥0.80)	1.75 (0.95, 3.23)		1.52 (0.82, 2.84)		1.20 (0.64, 2.23)	
Low-dose Intensity <0.70	Yes: 15	No: 221	Yes: 15	No: 202	Yes: 15	No: 175
HR (95% CI) (reference: dose intensity ≥0.70)	2.31 (1.01, 5.28)		3.36 (1.49, 7.55)		2.53 (1.10, 5.79)	

CI, confidence interval; HR, hazards ratio; RCC, renal cell carcinoma.

1 Each model was also adjusted for age at treatment initiation, number of metastatic sites, gender, time from initial RCC diagnosis to treatment, and country.

**Figure 1:**
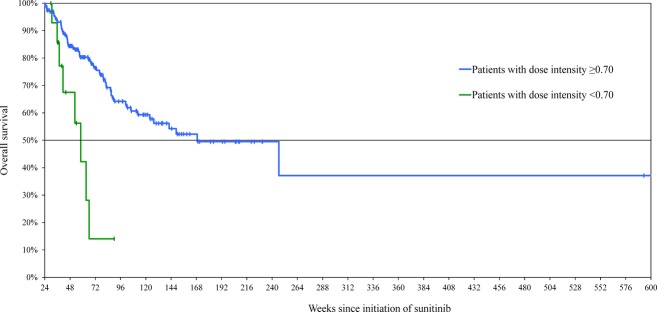
Kaplan–Meier estimates of overall survival by level of dose intensity (landmark period: 0–24 weeks).

The median overall survival for the 291 patients who had not died at the end of the 24-week landmark period was 168.9 weeks. Figure 1 shows the Kaplan–Meier estimates of OS by level of dose intensity during the 24-week landmark period. After adjusting for potential confounders, OS was significantly shorter among patients with a sunitinib dose intensity below 0.7 (Hazard Ratio [HR]: 3.36, 95% CI: [1.49, 7.55]), but dose intensities below 0.8 and 0.9 were not associated with significantly shorter survival times following the 24-week landmark period. Similarly, sunitinib low-dose intensity below 0.7 was associated with significantly shorter OS when considering landmark periods of 18 and 30 weeks, but no significant associations were observed between OS and dose intensities below 0.8 and 0.9 during the same landmark periods.

### Treatment modification and overall survival

Table 5 presents results from multivariate, Cox proportional hazard models quantifying the association between treatment modifications and OS. Of the 217 patients who were taking sunitinib and had not died at the end of the landmark period of 24 weeks, 12 (5.5%) had discontinued treatment, 69 (31.8%) had ≥1 dose reduction, and 44 (20.3%) had ≥1 treatment interruption. Overall, 80 (36.9%) patients had experienced at least one treatment modification due to AEs during the 24-week landmark period.

**Table 5:** Association between treatment modifications due to adverse events and time to death in patients with advanced RCC treated with sunitinib as first-line angiogenesis inhibitor treatment^1^.

	Treatment modification observation period (landmark period): 0–18 weeks		Treatment modification observation period (landmark period): 0–24 weeks		Treatment modification observation period (landmark period): 0–30 weeks	
			
Treatment modification during landmark period^2^	*N* = 236		*N* = 217		*N* = 195	
Discontinuation^3^	Yes: 14	No: 222	Yes: 12	No: 205	Yes: 14	No: 181
HR (95% CI) (reference: no discontinuation)	4.91 (2.29, 10.54)		2.80 (1.06, 7.38)		2.69 (1.05, 6.89)	
Dose reduction^4^	Yes: 64	No: 172	Yes: 69	No: 148	Yes: 70	No: 125
HR (95% CI) (reference: no dose reduction)	1.07 (0.63, 1.82)		1.28 (0.75, 2.20)		1.14 (0.65, 1.98)	
Dose Interruption^5^	Yes: 46	No: 190	Yes: 44	No: 173	Yes: 50	No: 145
HR (95% CI) (reference: no dose interruption)	1.50 (0.89, 2.52)		1.33 (0.77, 2.32)		1.61 (0.92, 2.82)	
Any treatment modification^6^	Yes: 78	No: 158	Yes: 80	No: 137	Yes: 85	No: 110
HR (95% CI) (reference: no treatment modification)	1.18 (0.72, 1.91)		1.26 (0.75, 2.09)		1.38 (0.81, 2.36)	

CI, confidence interval; HR, hazard ratio; RCC, renal cell carcinoma.

1 Each model was also adjusted for age at treatment initiation, number of metastatic sites, gender, time from initial RCC diagnosis to treatment, and country.

2 Includes patients who experienced a treatment modification and did not die or who were not censored at any point during the respective landmark period.

3 If a patient's discontinuation date was not available and the patient died, treatment duration was calculated from treatment initiation to date of death. If patient's discontinuation date was not available and there was no record of patient death, treatment duration was calculated from treatment initiation to date of last follow-up.

4 If a patient experienced multiple dose reductions, only the first dose reduction was accounted for.

5 If a patient experienced multiple dose interruptions, only the first dose interruption was accounted for.

6 If a patient experienced multiple treatment modification, only the first modification was accounted for.

After adjusting for potential confounders, overall survival was significantly shorter among patients who discontinued treatment due to AEs within 24 weeks of initiation (HR: 2.80, 95% CI: [1.06, 7.38]). Sunitinib treatment discontinuation within the first 18 and 30 weeks of therapy initiation was also associated with significantly shorter survival (18 weeks, HR: 4.91, 95% CI: [2.29, 10.54]; 30 weeks, HR: 2.69, 95% CI: [1.05, 6.89]). Other treatment modifications were not associated with significant decrease in survival after adjusting for potential confounders.

## Discussion

This study, which relied on data from medical charts from 291 treatment-naïve patients across five countries in Europe, contributes to the growing body of knowledge regarding the tolerability and management of side effects for patients receiving first-line anti-angiogenic agents for the treatment of advanced RCC [4, 5, 7–15]. The findings from this study indicate that treatment-naïve patients receiving first-line sunitinib experienced frequent AEs and high rates of treatment modifications within the first few months of treatment, leading to a suboptimal drug exposure. Given the high rates of AEs reported among patients taking sunitinib, the relationship between AEs and treatment modifications, and the resulting observed association on dose intensity and overall survival are important considerations.

These findings are consistent with results from a pharmacokinetic/pharmacodynamic meta-analysis investigating the relationship between exposure to sunitinib and efficacy and tolerability endpoints in patients with cancer, including advanced RCC [16]. The authors found that increased exposure to sunitinib was associated with longer time to progression, longer OS, a higher probability of a response, and greater tumor size decreases, thus recognizing the importance of avoiding unscheduled dose titrations and treatment interruptions. Increased exposure to sunitinib was also associated with AEs, although these were generally mild to moderate in severity [16]. Furthermore, the Renal EFFECT trial found no benefit in efficacy or safety for sunitinib 37.5 mg/day given on a continuous daily dosing compared with the approved intermittent schedule of 50 mg/day given on 4/2 cycles—corresponding to a lower daily dosing of 33.3 mg—in patients with advanced RCC [17]. The lack of recovery time from sunitinib-related AEs could be hypothesized as a factor explaining the absence of benefit in efficacy associated with the increased exposure to sunitinib offered by the 37.5 mg continuous daily dosing.

The method used for this analysis determined rates of specific AEs and treatment patterns following a particular exposure of interest during a given period (landmark period) following treatment initiation. Thus, results are difficult to compare with that of the respective sunitinib RCT and EAP [4, 5]. One would expect lower rates of reported AEs in an observational study. Furthermore, by design, only AEs among patients who lived until the end of the landmark period are reported in the current study. Similarly, RCTs and EAPs have well-defined protocol for dose reduction as well as treatment interruption and discontinuation which may lead to distinctive treatment patterns compared to those observed in real-world practice, thus affecting dose intensity. In an EAP, the mean (SD) relative dose intensity of sunitinib was 0.952 (0.253) [5], which is higher than the mean dose intensity observed in the current study beyond the AE observation period of 24 weeks (no AE: 0.845 [0.279; ≥1 AE: 0.837 [0.250]).

Regional differences in the availability of alternative treatment options may influence sunitinib treatment patterns and dose intensity and may have contributed to variation within the current study's population. For example, in the UK, until 2010 sunitinib was the only drug recommended for treatment of advanced RCC by the National Institute for Health and Clinical Excellence and most primary care trusts did not fund other drugs for advanced RCC [8]. In comparison, sunitinib, sorafenib, and bevacizumab were available and used in France and Ireland at the time of the chart review abstraction. As such, the proportion of patients experiencing sunitinib treatment discontinuation was markedly lower in the UK compared with France and Ireland (UK: 43.7%; France: 69.9%; Ireland: 69.8%) while the opposite trend was observed for dose reduction (UK: 48.7%; France: 43.9%; Ireland: 34.0%) and treatment interruption (UK: 37.8%; France: 9.2%; Ireland: 35.8%). Corroborated by the longer median treatment duration observed in the UK (months, UK: 19.8; France 10.7; Ireland: 8.7), these results suggest that physicians in the UK may try to keep patients on sunitinib for as long as possible while managing adverse reactions with dose reductions and treatment interruption given the lack of alternative options at the time. Thus, by staying longer on sunitinib with more dose reductions and interruptions, patients from the UK (N: 119; 40.9%) may have contributed to lower the average dose intensity of the study population.

The sample sizes for each country were too small to conduct individual country-level analyses. With more data such analyses may have been of interest as patients in the UK, for example, were likely to maintain sunitinib treatment for longer for reasons stated above. This longer treatment duration could influence associations between AEs and dose intensity, dose intensity and survival, and treatment modification and survival. Despite not conducting individual country-level analyses, all multivariate models in the current study were adjusted for country. The specific results reported in this manuscript show the measure of association and 95% CI for the main exposure of interest and not for each factor included in the model. However, when we examine the coefficients associated with each country indicator for the analysis of the association between AEs assessed at 24 weeks and low-dose intensity we see that there are no significant associations between specific countries and the outcome in the model (low-dose intensity). No significant trend in country-specific effects emerged in the other analyses as well.

This study raises questions regarding optimal dose intensity which may be answered in future studies. The current study examined the sequential association of AEs during the early part of treatment (landmark period) and subsequent dose intensity, and separately, the sequential association of dose intensity during the early part of treatment (landmark period) and death. Thus, with the current data and analysis it is not possible to determine the particular time point following an AE when the dose intensity falls below a dosing point that compromises survival. However, it was determined in the current analysis that the inflection point for survival was somewhere between 70% and 80% dose intensity. A sensitivity analysis was conducted to assess the association between low-dose intensity during the landmark period and subsequent survival for those patients who later did and did not discontinue sunitinib treatment as a way to evaluate the impact of disease dissemination on survival. The association between low-dose intensity (<0.70) and survival remained significant for patients who did later discontinue sunitinib and was no longer significant for those who did not later discontinue sunitinib. This finding may indicate that survival differences by dose intensity during the landmark period may be attributable to disseminated disease subsequent to the suboptimal dose intensity as patients with disseminated disease may be the ones to discontinue therapy.

Furthermore, future studies may consider additional clinical endpoints of interest such as disease progression and progression-free survival (PFS). The data for the current study came from real-world, clinical practice, and disease progression is not systematically measured and reported in these settings, as it is in clinical trials. Thus, in this context, the observed PFS is more a function of when progression was assessed rather than when the patient actually progressed. For that reason, the current study does not assess PFS as an outcome. RCTs and EAPs, which capture outcomes such as PFS systematically, have analyzed progression or PFS as the primary endpoint for first-line treatment with sunitinib for advanced RCC as subsequent-line therapies affect OS [3–5, 17]. Second-line therapies may have had an impact on the OS reported in the current study as 18% of the patients included in the main analysis of the impact of dose intensity on OS received second-line therapy. Patients with a dose intensity less than 0.70 were more likely than those with greater dose intensity to receive second-line therapy (33% vs. 17%), so it is possible that the difference in second-line therapy between those with low and non-low first-line sunitinib dose intensity contributed to the observed difference in survival by dose intensity. It is also possible that patients with low density for first-line therapy could have been more like to progress, and therefore switch from first-line to second-line therapy, and the progression may be associated with shorter survival.

There are some limitations associated with this study. This study used retrospectively collected data; occurrences of AEs, treatment modifications, and outcome may have been underreported. The reported AEs were likely to be clinically significant AEs that required interventions. Real-world clinical practice settings are likely to capture fewer AEs in medical records compared with RCT settings which have systematic procedures to capture AEs. If the medical records in the current study were more likely to include AEs which led to treatment modifications lowering dose intensity then this could have biased the results such that the association between AEs and lower dose intensity is overestimated. Given the low number of patients experiencing certain exposures of interest during the landmark periods, there may have been insufficient power to detect differences in outcomes between groups in some analyses. It is not known whether any patients in this study received sunitinib and interferon simultaneously. While this may have been unlikely, we cannot preclude the possibility as the inclusion criteria were such that patients received sunitinib as first-line therapy and had no prior interferon. If some patients did receive interferon treatment this may have contributed to some AEs and associated treatment modifications, reducing dose intensity. In addition, due to low sample counts, the analyses did not distinguish between specific types of AEs, such as fatigue, hand–foot syndrome, mucositis/stomatitis, and their association with dose intensity. Also, adjusted analyses did not control for baseline Eastern Cooperative Oncology Group (ECOG) performance score as a large proportion of patients did not have baseline scores available (≥55%). It should also be noted that this study was performed using data collected from 10 tertiary oncology centers in France, Ireland, Italy, Spain, and UK; results may not be generalizable to community oncology centers or other European countries where clinical practice may differ. Furthermore, while these analyses adjusted for known confounders, imbalances in unobserved characteristics influencing survival may be present, and these were not adjusted for. Unobserved characteristics include Memorial Sloan-Kettering Cancer Center (MSKCC) risk groups, and prognostic factors reported by Heng et al. [18]. such as hemoglobin and calcium levels, and neutrophil and platelet counts, Finally, as this is an observational study, it is important to stress that only statistical correlations could be established which by no mean infers the existence of causal relations.

This study provided evidence through a comprehensive review of medical charts for 291 advanced RCC patients across key institutions in five European countries that severe AEs were significantly correlated with lower dose intensities, and that AEs were frequently cited as reasons for treatment modifications and discontinuations. This study further showed that low-dose intensities and treatment discontinuations were correlated with shorter survival times, indicating the importance of maintaining dose intensity. These results suggest that the use of agents with a better tolerability profile coupled with proper therapy management including prevention, early recognition, and prompt management of AEs may be important for patients with advanced RCC.
